# The screen for cognitive impairment in psychiatry: diagnostic-specific standardization in psychiatric ill patients

**DOI:** 10.1186/1471-244X-13-127

**Published:** 2013-05-06

**Authors:** Juana Gómez-Benito, Georgina Guilera, Óscar Pino, Emilio Rojo, Rafael Tabarés-Seisdedos, Gemma Safont, Anabel Martínez-Arán, Manuel Franco, Manuel J Cuesta, Benedicto Crespo-Facorro, Miguel Bernardo, Eduard Vieta, Scot E Purdon, Francisco Mesa, Javier Rejas

**Affiliations:** 1 Department of Methodology, Faculty of Psychology, University of Barcelona, Barcelona, Spain; 2 Institute for Brain, Cognition, and Behavior (IR3C), University of Barcelona, Barcelona, Spain; 3 Department of Psychiatry, Benito Menni CASM, Granollers Hospital General, Granollers, Barcelona, Spain; 4 Department of Medicine, Teaching Unit of Psychiatry and Psychological Medicine, University of Valencia, CIBERSAM, Valencia, Spain; 5 Schizophrenia Program Clinic, Institute of Neuroscience, Hospital Clinic i Provincial, IDIBAPS, University of Barcelona, CIBERSAM, Barcelona, Spain; 6 Bipolar Disorders Program, Institute of Neuroscience, Hospital Clinic i Provincial, IDIBAPS, CIBERSAM, University of Barcelona, Barcelona, Spain; 7 Department of Psychiatry, Hospital Provincial Rodríguez Chamorro, Zamora, Spain; 8 Psychiatric Hospitalization Unit, Hospital Virgen del Camino, Pamplona-Iruña, Spain; 9 Department of Psychiatry, Hospital University Marqués de Valdecilla, Santander, Spain; 10 Department of Psychiatry, Bebensee Schizophrenia Research Unit, University of Alberta, Edmonton, AB, Canada; 11 Department of Neurosciences, Medical Unit, Pfizer Spain, Alcobendas, Madrid, Spain; 12 Health Outcomes Research Department, Medical Unit, Pfizer Spain, Alcobendas, Madrid, Spain

**Keywords:** SCIP-S, Standardization data, Norms, Schizophrenia, Bipolar I disorder

## Abstract

**Background:**

The Screen for Cognitive Impairment in Psychiatry (SCIP) is a simple and easy to administer scale developed for screening cognitive deficits. This study presents the diagnostic-specific standardization data for this scale in a sample of schizophrenia and bipolar I disorder patients.

**Methods:**

Patients between 18 and 55 years who are in a stable phase of the disease, diagnosed with schizophrenia, schizoaffective disorder, schizophreniform disorder, or bipolar I disorder were enrolled in this study.

**Results:**

The SCIP-S was administered to 514 patients (57.9% male), divided into two age groups (18–39 and 40–55 years) and two educational level groups (less than and secondary or higher education). The performance of the patients on the SCIP-S is described and the transformed scores for each SCIP-S subtest, as well as the total score on the instrument, are presented as a percentile, z-score, T-scores, and IQ quotient.

**Conclusions:**

We present the first jointly developed benchmarks for a cognitive screening test exploring functional psychosis (schizophrenia and bipolar disorder), which provide increased information about patient’s cognitive abilities. Having guidelines for interpreting SCIP-S scores represents a step forward in the clinical utility of this instrument and adds valuable information for its use.

## Background

Cognitive deficits are highly prevalent in psychotic disorders [1], including schizophrenia, bipolar disorder, and schizoaffective disorder [2-6]. Numerous studies suggest that patients with severe psychiatric disorders have impaired sustained attention [7] and memory [8-10]. A wide spectrum of executive deficits have also been described, including problems performing goal-oriented tasks, recognizing priority patterns, and planning [11,12], along with diminished verbal fluency [13] and information processing speed [14,15]. Increasing recognition that psychosocial prognosis is directly related to the severity of the cognitive impairments [16-19], has resulted in a paradigm shift that may expand the targets for treatment beyond the mere symptom suppression and necessitate an integration of cognitive assessment into routine psychiatric practice.

The importance on the field of this study is emphasized by a long-standing initiative of the National Institute of Mental Health, known as MATRICS [20,21], which has now been subdivided into three different programs: CNTRICS [22], TURNS [23], and TENETS (Treatment and Evaluation Network for Trials in Schizophrenia). The aim of these initiatives is to unify and standardize the types of deficits to be measured and the tests to use, with the final objective of developing effective new treatments for the neurocognitive deficits that occur in these patients. Recently, the MATRICS initiative has proposed a consensus battery that takes between 60 and 90 minutes to administer and is composed of 10 paper-and-pencil tests specifically for cognitive assessment of patients with schizophrenia – the MATRICS Consensus Cognitive Battery [24,25]. Given the difficulties of performing an assessment lasting more than one hour in standard clinical practice, in the past few decades, considerable effort has been made to create brief cognitive batteries that facilitate an overall understanding of the individual’s cognitive status, without overly sacrificing the sensitivity and specificity of these new instruments. Some examples are the Cognistat, before 1995 known as the Neurobehavioral Cognitive Status Examination [26], the Repeatable Battery for the Assessment of Neuropsychological Status (RBANS) [27], the Woodcock-Johnson III Test of Cognitive Abilities (WJ III COG) [28], and the Brief Assessment of Cognition in Schizophrenia (BACS) [29]. These instruments have decreased the time it takes to assess patients to about 40–50 minutes, but even so they have a high cost in terms of time and economics due to time constraints on practitioners in their daily clinical practice.

More recently, other types of studies have focused on the development of cognitive screening tools – scales that do not require additional materials in order to be administered, tools that have different interchangeable versions, tools that are simple and easy to administer, and have an administration time that is appropriate and manageable in clinical practice, i.e., approximately 15 minutes. Some examples are the Brief Cognitive Assessment (BCA) [30], the Screen for Cognitive Impairment in Psychiatry (SCIP) [31], and the Brief Cognitive Assessment Tool for Schizophrenia (B-CATS) [32]. All of these have good psychometric properties [30-35], but still no standardization data have been established for any of them.

A Spanish translation of the SCIP was recently introduced (SCIP-S) [36] which demonstrated appropriate psychometric properties both for patients with a diagnosis of schizophrenia [34] and those with bipolar I disorder [33], with regard to equivalence between parallel forms, internal consistency, temporal stability, dimensional structure, and convergent validity. Tentative cut-scores for identification of significant cognitive impairment irrespective of diagnosis are available [35], but the resulting binary classification is insufficient for description of the severity of identified impairment relative to a patient’s clinical cohort after adjustment for age, gender, and education. Guidelines for the interpretation of the SCIP-S would thus represent a step forward in the clinical utility of this instrument and add valuable information on its proper use.

Normative data represent performance on a measure or test by a standardization sample against which other performances on the measure can be compared [37]. Lack of normative data limits the interpretation of scores in individual cases as well as in treatment outcome research (as we cannot know if a score is typical, high or low for the population being studied) [38]. This has implications for our ability to assess the clinical significance of a score (or change in a score). Norm scores can assist clinicians in providing quantitative labels for the degree to which a raw score is to be considered average, elevated, or extreme and might be useful for diagnostic purposes, clinical decision making, or evaluation of treatment effects [37]. A traditional approach to deriving norm scores is to compare an individual’s raw score to a reference group with the same condition matched for background variables such as age and gender. In addition, clinicians using norms for comparison can more readily interpret a patient’s performance on a number of relevant self-report dimensions as well. This should assist in the determination of whether or not an individual’s responses are unusual for someone experiencing, in this case, cognitive deficits. In turn, this may suggest possible courses of action, such as further investigation or treatment (whose outcome can be evaluated against the normative dataset) [37,38].

When evaluating cognitive function in routine practice, clinicians usually compare the patient score against the norms in the general healthy population to ascertain whether the patient cognitive function is preserved or impaired. In such case, comparison allows to determine the distance to what a particular patient separate from the mean score. Nevertheless, in many cases the practitioner refers the patient to a specialist for formal recognition when his/her performance is unusually low compared with patients with same condition. Particularly when additional etiologies (in addition or replacement of schizophrenia) responsible for the high cognitive impairment observed is suspected [39]. Concerning this point, patient score should be compared with the norms belonging to subjects with the same health condition to assess how the different is the patient scoring related to his/her population of reference. As stated by Irvison et al. [40], this information can improve the clinician´s understanding of patient´s cognitive strength and weakness, put a patient’s cognitive abilities into perspective for their diagnosis, and facilitate multidisciplinary treatment decisions.

In this context, to date there are no standardization data for the SCIP-S scale in psychiatric patients that allow the examiner to interpret the patient scores relative to the cognitive performance of their peers. Thus, the objective of this study is to provide the first clinical normative data for the SCIP-S in patients with functional psychosis, and specifically with schizophrenia-spectrum disorder or bipolar I disorder.

## Methods

### Participants

Patients diagnosed criteria with schizophrenia, schizoaffective disorder, schizophreniform disorder, or bipolar I disorder according to DSM-IV-TR [41] were enrolled in this study. To take part, patients had to be between 18 and 55 years of age and in a stable phase of the disease. In the case of patients with schizophrenia spectrum disorders, stability was defined by: a) no hospitalization in the past 3 months, and b) a total score of less than 70 on the Positive and Negative Syndrome Scale (PANSS) [42,43]. In the case of patients with bipolar I disorder, stability was defined as: a) 6 or more months in remission, b) a score less than or equal to 7 on the 17-items Hamilton Depression Scale (HAM-D) [44], and c) a score less than or equal to 6 on the Young Mania Rating Scale (YMRS) [45]. We excluded individuals that were participating in a clinical trial, and those with a serious medical or neurological condition, another psychiatric disorder as a primary diagnosis or main reason for treatment, major depression, or difficulty reading and/or writing. The process of recruitment began with a consensus conference on the diagnostic criteria for the different schizophrenia spectrum disorders and the bipolar disorder I. This consensus was adopted by all participating psychiatrists. This conference dealt mainly with the standard psychiatric interview based on the DSM-IV diagnostic criteria (anamnesis and the exploration of the mental condition), the PANSS, HAM-D, and YMRS scales, and the different inclusion/exclusion criteria of our study.

### Instrument

The SCIP [31] is a brief screening tool designed to assess cognitive impairment in psychiatric patients. It has five subtests for evaluating immediate (Verbal Learning Test-Immediate; VLT-I) and delayed (Verbal Learning Test-Delayed; VLT-D) verbal learning, working memory (Working Memory Test; WMT), verbal fluency (Verbal Fluency Test; VFT), and processing speed (Processing Speed Test; PST). It may be administered without the need for additional equipment, i.e., a pencil and a stopwatch, and requires nearly 15 min. Three alternative forms of the scale are available to facilitate repeated testing. Table 1 contains the description and the main characteristics of the SCIP subtests.

**Table 1:** Description of the SCIP subtests

**Subtest**	**Description**	**Score**	**Range scores**
VLT-I	Three trials of a 10 word list-learning task with immediate recall after each list presentation	Sum of the number of words correctly recalled over the three trials	0-30
WMT	Eight 3-letter combinations of consonants, with two trigrams each assigned to a 0, 3, 9, or 18 second delay with backward counting distraction.	Sum of the letters correctly recalled	0-24
VFT	Two trials of 30 seconds during which the subject is asked to generate words that begin with a given letter of the alphabet under some specific rules	Sum of acceptable words over the two trials	≥ 0
VLT-D	Delayed recall test of the VLT-I words	Sum of the number of words correctly recalled	0-10
PST	Task that in 30 seconds requires the subject to translate the Morse code equivalents of six letters from the alphabet in boxes under a randomly distributed sequence of the letters	Sum of the number of correct sequential translations	0-30

VLT-I = Verbal Learning Test-Immediate; WMT = Working Memory Test; VFT = Verbal Fluency Test; VLT-D = Verbal Learning Test-Delayed; PST = Processing Speed Test.

The psychometric properties of the SCIP were studied in a sample of patients with schizophrenia [34] and in a sample of bipolar I patients [33], and were shown to be adequate. Specifically, both studies demonstrated the equivalence among the three parallel forms, internal consistency (Cronbach’s alpha of 0.73 and 0.74, respectively), and test-retest reliability (intraclass correlation coefficient of 0.90 and 0.87, respectively, for the SCIP total score). Convergent validity was supported by the associations between SCIP subtests and conventional neuropsychological instruments applied in routine clinical practice. The scores also converged on a single cognitive factor accounting for around 50% of the total variance, suggesting a one-factor internal structure in both samples named cognitive performance. Finally, when comparing cognitively-impaired individuals and those with adequate functioning, the proposed cut-off point of the SCIP (< 70) was associated with a sensitivity of 87.9 and specificity of 80.6.

### Procedure

This study was approved by the University of Barcelona Ethics Committee. The SCIP-S was administered to all patients, who were systematically tested once it was confirmed that they met the study inclusion criteria and gave their written informed consent to voluntarily participate in the study; data confidentiality was maintained at all times. The data were collected at 119 Spanish mental health centers, selected by probability sampling adjusted by population weights from the 17 Spanish Autonomous Communities, with the participation of 132 psychiatrists duly trained in administering the instrument with a video designed for that purpose. Before the start of the process, a neuropsychologist experienced in administration of neuropsychological tests and batteries trained a sub-set of forty-four psychiatrists in a 60-minute session to ensure consistency in SCIP administration and correction. The training phase was completed with a kappa index of agreement in scale correction and scoring of .99.

### Data analysis

The analyses were done using the SPSS statistical package version 15 and the significance level was set at α = .01. The internal consistency of the SCIP was assessed by computing Cronbach’s alpha coefficient, treating each of the SCIP subtests as variables. We compared the SCIP scores of patients with schizophrenia and bipolar I disorder, as well as between males and females, using a *t* test for independent samples. In both cases, the statistical significance was supplemented by calculating Cohen’s *d*. Likewise, the differences between the specified groups were analyzed by age and educational level. The normal distribution of the data was tested using the Kolmogorov-Smirnov (KS) test for normality.

Patient performance on the SCIP was shown using various descriptive statistics (mean, standard deviation, median, asymmetry, kurtosis, and range of scores). As for the transformation of SCIP scores, a percentile, z-score, T-scores (*T* = 50 + 10 » *z*), and intelligence quotient (*IQ* = 100 + 15 » *z*) were calculated.

## Results

### Sample description

A total of 514 patients diagnosed according to DSM-IV-TR [41] criteria with schizophrenia (41.5%), schizoaffective disorder (6.4%), schizophreniform disorder (1.4%), or bipolar I disorder (50.7%) participated in this study. Within this group, 57.9% were males. Most patients with schizophrenia were being treated with a single antipsychotic (66.9%), although a large number were receiving a combination of two (28.0%) or three (3.1%) antipsychotics. At the time of assessment, 5 patients were not taking any antipsychotic. In addition to the antipsychotic medication, 52.6% of patients were receiving an additional psychoactive drug, primarily antidepressants and benzodiazepines. The mean age at onset of the illness was 24.25 (SD = 6.34), the mean number of months since the diagnosis was 156.78 (102.99), and the mean number of hospitalizations was 2.61 (3.05). Within the bipolar I disorder sample, 23.5% were taking lithium, while other patients were taking one (33.5%) or two (3.5%) antipsychotics in addition to lithium, and finally another group of patients were taking receiving antipsychotic medication in monotherapy (23.8%), or in a combination of two (5.0%), or three (0.4%) agents. Additionally, 75.4% of patients were receiving another type of psychoactive drug (i.e., antidepressants or benzodiazepines). The mean age at onset of the illness was 28.39 (8.34), the mean number of months since the diagnosis was 144.55 (95.78), the mean number of manic episodes they had experienced was 3.36 (1.86), and of depressive episodes was 1.22 (2.94), and finally the mean number of hospitalizations was 2.80 (3.67).

The participants were divided into two age groups (18–39 and 40–55) and two education level groups (less than secondary education and secondary education or higher). Based on these and other variables, Table 2 shows the main demographic information for each clinical sample used in the study.

**Table 2:** Sample descriptors

**Variable (Percentage)**	**Schizophrenia (n = 254)**	**Bipolar I disorder (n = 260)**
Sex		
Male	71.3	44.8
Female	28.7	55.2
Educational level		
< Secondary education	33.9	33.5
≥ Secondary education	66.1	66.5
Age		
18 – 39	58.7	45.8
40 – 55	41.3	54.2

### Comparison between groups

By comparing the scores of patients with schizophrenia and bipolar disorder I, as well as between men and women, we obtained the means, standard deviations, *t* statistics, and effect sizes specified in Table 3.

**Table 3:** Mean SCIP scores and standard deviations by clinical diagnosis and sex of patients

	**Diagnosis**				**Sex**			
**Subtest**	**Schizophrenia**	**Bipolar I**	***t *test**	***d***	**Male**	**Female**	***t *test**	***d***
VLI-I	18.83 (4.07)	19.47 (4.10)	t(512) = 1.752	0.16	18.90 (3.97)	19.47 (4.22)	t(511) = 1.562	0.14
WMT	17.10 (4.46)	17.61 (4.20)	t(512) = 1.344	0.12	17.70 (4.26)	16.88 (4.40)	t(511) = 2.125	0.19
VFT	15.13 (5.74)	15.83 (5.83)	t(511) = 1.371	0.12	15.28 (5.55)	15.67 (5.98)	t(510) = 0.761	0.07
VLT-D	5.17 (2.35)	5.63 (2.34)	t(511) = 2.240	0.20	5.09 (2.38)	5.81 (2.24)	t(510) = 3.469*	0.31
PST	9.25 (3.54)	9.80 (3.49)	t(509) = 1.771	0.16	9.48 (3.58)	9.59 (3.46)	t(580) = 0.330	0.03
SCIP Total	65.50 (14.41)	68.20 (14.32)	t(507) = 2.120	0.19	66.39 (13.67)	67.37 (15.27)	t(506) = 0.755	0.07

* *p* < .01.

Both in the various subtests and in the total score, the mean performance of the patients with schizophrenia was poorer than that of the patients with bipolar I disorder, although in no case was the effect size measurement significant. With respect to sex, the mean scores were similar, with the exception of the VLT-D subtest, where women scored slightly better than men, although the corresponding effect size was small. As was to be expected, on all subtests, as well as on the total SCIP score, the patients with a higher education scored higher than those with a less than secondary education (all *p* values < .01), with effect sizes that varied between 0.50 for the VFT subtest and 0.70 for the PST subtest. The difference in total SCIP score, for the education variable, reached an effect size of 0.78. In the case of age, as the patients’ age increases, the mean scores decreased. The differences were statistically significant (*p* value < .01) for the total SCIP score and the various subtests, with the exception of the VLT-I and VFT. The effect sizes varied between 0.25 for the VLT-I subtest and 0.54 for the PST subtest, while the difference in total SCIP score was characterized by having an effect size of 0.53. For those reasons, the clinical normative benchmarks are presented jointly for male and female patients with schizophrenia and bipolar I disorder. On the other hand, given the existing differences, patient age and educational level were taken into account.

### Standardization data

The internal consistency of the SCIP achieved a Cronbach’s alpha coefficient of 0.73, which is an acceptable value given the small number of variables. This alpha value did not increase when any of the component variables were eliminated. The item/scale correlations were between 0.44 for the VFT and 0.58 for the PST. The normal distribution of the data from the various subtests (and total SCIP-S score) was tested for each of the groups after combining the two age groups and the two participant educational level groups. In no case was the KS test statistically significant at a level of 0.01 (all *p* > .01) although in six cases there were *p* values below .05 (the WMT, VFT, VLT-D, and PST subtests in the 18–39 year old group and the VLT-D and PST subtests in the 40–55 year old group, in all cases with a secondary or higher education). Tables 4, 5, 6, 7 and 8 show the clinical normative data for each of the SCIP-S subtests in terms of percentiles, z-scores, T-scores, and IQ. Likewise, Table 9 shows this same information for the total SCIP-S score.

**Table 4:** Transformation of VLT-I subtest scores

**< Secondary school**										**≥ Secondary school**									
**18-39**					**40-55**					**18-39**					**40-55**				
**PD**	**P**	**z**	**T**	**IQ**	**PD**	**P**	**z**	**T**	**IQ**	**PD**	**P**	**z**	**T**	**IQ**	**PD**	**P**	**z**	**T**	**IQ**
0-11	< 3	< −1.72	< 33	< 74	0-9	1	< −2.19	< 28	< 67	0-5	1	< −3.45	< 16	< 48	0-9	1	< −2.4	< 26	< 64
0-11	< 3	< −1.72	< 33	< 74	0-9	1	< −2.19	< 28	< 67	6	1	−3.45	16	48	0-9	1	< −2.4	< 26	< 64
0-11	< 3	< −1.72	< 33	< 74	0-9	1	< −2.19	< 28	< 67	7	1	−3.21	18	52	0-9	1	< −2.4	< 26	< 64
0-11	< 3	< −1.72	< 33	< 74	9	1	−2.19	28	67	8	1	−2.96	20	56	0-9	1	< −2.4	< 26	< 64
0-11	< 3	< −1.72	< 33	< 74	10	2	−1.93	31	71	9	1	−2.72	23	59	10	1	−2.4	26	64
0-11	< 3	< −1.72	< 33	< 74	11	5	−1.67	33	75	10	1	−2.48	25	63	11	2	−2.14	29	68
12	3	−1.72	33	74	12	9	−1.41	36	79	11	3	−2.24	28	66	12	3	−1.89	31	72
13	9	−1.45	35	78	13	15	−1.15	39	83	12	4	−1.99	30	70	13	5	−1.63	34	76
14	13	−1.18	38	82	14	22	−0.89	41	87	13	5	−1.75	33	74	14	9	−1.37	36	79
15	19	−0.9	41	86	15	28	−0.62	44	91	14	8	−1.51	35	77	15	15	−1.12	39	83
16	30	−0.63	44	91	16	33	−0.36	46	95	15	10	−1.26	37	81	16	22	−0.86	41	87
17	43	−0.36	46	95	17	44	−0.10	49	98	16	14	−1.02	40	85	17	28	−0.61	44	91
18	51	−0.08	49	99	18	56	0.16	52	102	17	19	−0.78	42	88	18	36	−0.35	46	95
19	57	0.19	52	103	19	64	0.42	54	106	18	26	−0.54	45	92	19	46	−0.10	49	99
20	63	0.46	55	107	20	72	0.68	57	110	19	37	−0.29	47	96	20	54	0.16	52	102
21	70	0.73	57	111	21	81	0.94	59	114	20	47	−0.05	49	99	21	61	0.41	54	106
22	80	1.01	60	115	22	89	1.20	62	118	21	56	0.19	52	103	22	73	0.67	57	110
23	88	1.28	63	119	23	94	1.47	65	122	22	66	0.43	54	107	23	82	0.93	59	114
24	94	1.55	66	123	24	97	1.73	67	126	23	75	0.68	57	110	24	88	1.18	62	118
25	98	1.83	68	127	25	98	1.99	70	130	24	82	0.92	59	114	25	94	1.44	64	122
26-30	99	> 1.83	> 68	> 127	26	99	2.25	72	134	25	87	1.16	62	117	26	96	1.69	67	125
26-30	99	> 1.83	> 68	> 127	27	99	2.51	75	138	26	93	1.41	64	121	27	98	1.95	69	129
26-30	99	> 1.83	> 68	> 127	28-39	99	> 2.51	> 75	> 138	27	97	1.65	66	125	28	99	2.20	72	133
26-30	99	> 1.83	> 68	> 127	28-39	99	> 2.51	> 75	> 138	28	98	1.89	69	128	29	99	2.46	75	137
26-30	99	> 1.83	> 68	> 127	28-39	99	> 2.51	> 75	> 138	29	99	2.13	71	132	30	99	> 2.46	> 75	> 137
26-30	99	> 1.83	> 68	> 127	28-39	99	> 2.51	> 75	> 138	30	99	2.38	74	136	30	99	> 2.46	> 75	> 137
N	64				N	109				N	204				N	137			
Mean	18.31				Mean	17.39				Mean	20.21				Mean	19.38			
SD	3.660				SD	3.829				SD	4.120				SD	3.913			
Median	18				Median	17				Median	20				Median	19			
Skewness	0.048				Skewness	−0.045				Skewness	−0.378				Skewness	−0.125			
Kurtosis	−1,023				Kurtosis	−0.456				Kurtosis	0.340				Kurtosis	−0.380			
Range	12-25				Range	9-27				Range	6-30				Range	10-29			

**Table 5:** Transformation of WMT subtest scores

**< Secondary school**										**≥ Secondary school**									
**18-39**					**40-55**					**18-39**					**40-55**				
**PD**	**P**	**z**	**T**	**IQ**	**PD**	**P**	**z**	**T**	**IQ**	**PD**	**P**	**z**	**T**	**IQ**	**PD**	**P**	**z**	**T**	**IQ**
0-1	1	< −3.25	< 17	< 51	0-4	1	< −2.40	< 26	< 64	0-5	1	< −3.27	< 17	< 51	0-3	1	< −3.19	< 18	< 52
2	1	−3.25	17	51	0-4	1	< −2.40	< 26	< 64	0-5	1	< −3.27	< 17	< 51	0-3	1	< −3.19	< 18	< 52
3	2	−3.04	20	54	0-4	1	< −2.40	< 26	< 64	0-5	1	< −3.27	< 17	< 51	0-3	1	< −3.19	< 18	< 52
4	2	−2.82	22	58	0-4	1	< −2.40	< 26	< 64	0-5	1	< −3.27	< 17	< 51	4	1	−3.19	18	52
5	2	−2.61	24	61	5	1	−2.40	26	64	0-5	1	< −3.27	< 17	< 51	5	1	−2.95	20	56
6	2	−2.39	26	64	6	1	−2.16	28	68	6	1	−3.27	17	51	6	1	−2.72	23	59
7	2	−2.18	28	67	7	1	−1.91	31	71	7	1	−3.01	20	55	7	2	−2.48	25	63
8	4	−1.97	30	71	8	3	−1.67	33	75	8	1	−2.75	22	59	8	3	−2.25	28	66
9	5	−1.75	32	74	9	8	−1.43	36	79	9	1	−2.49	25	63	9	3	−2.01	30	70
10	6	−1.54	35	77	10	13	−1.19	38	82	10	3	−2.23	28	67	10	4	−1.78	32	73
11	11	−1.32	37	80	11	18	−0.95	40	86	11	5	−1.97	30	70	11	7	−1.54	35	77
12	17	−1.11	39	83	12	27	−0.71	43	89	12	6	−1.71	33	74	12	10	−1.31	37	80
13	21	−0.89	41	87	13	35	−0.47	45	93	13	9	−1.45	36	78	13	14	−1.07	39	84
14	24	−0.68	43	90	14	43	−0.23	48	97	14	13	−1.19	38	82	14	19	−0.84	42	87
15	28	−0.46	45	93	15	52	0.01	50	100	15	18	−0.93	41	86	15	26	−0.60	44	91
16	34	−0.25	48	96	16	61	0.25	53	104	16	25	−0.67	43	90	16	35	−0.37	46	94
17	42	−0.03	50	99	17	67	0.49	55	107	17	32	−0.41	46	94	17	43	−0.13	49	98
18	48	0.18	52	103	18	71	0.73	57	111	18	39	−0.15	49	98	18	51	0.10	51	102
19	59	0.39	54	106	19	79	0.98	60	115	19	50	0.11	51	102	19	61	0.34	53	105
20	70	0.61	56	109	20	87	1.22	62	118	20	60	0.38	54	106	20	69	0.57	56	109
21	79	0.82	58	112	21	93	1.46	65	122	21	70	0.64	56	110	21	76	0.81	58	112
22	86	1.04	60	116	22	97	1.70	67	125	22	79	0.90	59	113	22	83	1.04	60	116
23	92	1.25	63	119	23	98	1.94	69	129	23	87	1.16	62	117	23	89	1.28	63	119
24	98	1.47	65	122	24	99	2.18	72	133	24	96	1.42	64	121	24	96	1.51	65	123
N	64				N	204				N	204				N	137			
Mean	17.16				Mean	14.95				Mean	18.56				Mean	17.57			
SD	4.661				SD	4.153				SD	3.838				SD	4.258			
Median	18				Median	15				Median	19				Median	18			
Skewness	−0.811				Skewness	0.020				Skewness	−0.625				Skewness	−0.549			
Kurtosis	0.550				Kurtosis	−0.746				Kurtosis	−0.036				Kurtosis	0.171			
Range	2-24				Range	5-24				Range	6-24				Range	4-24			

**Table 6:** Transformation of VFT subtest scores

**< Secondary school**										**≥ Secondary school**									
**18-39**					**40-55**					**18-39**					**40-55**				
**PD**	**P**	**z**	**T**	**IQ**	**PD**	**P**	**z**	**T**	**IQ**	**PD**	**P**	**z**	**T**	**IQ**	**PD**	**P**	**z**	**T**	**IQ**
0-4	1	< −1.79	< 32	< 73	0-1	1	< −1.79	< 32	< 73	0-6	1	< −1.74	< 33	< 74	0-3	1	< −2.22	< 28	< 67
0-4	1	< −1.79	< 32	< 73	2	1	−1.79	32	73	0-6	1	< −1.74	< 33	< 74	0-3	1	< −2.22	< 28	< 67
0-4	1	< −1.79	< 32	< 73	3	2	−1.63	34	76	0-6	1	< −1.74	< 33	< 74	0-3	1	< −2.22	< 28	< 67
0-4	1	< −1.79	< 32	< 73	4	5	−1.46	35	78	0-6	1	< −1.74	< 33	< 74	4	1	−2.22	28	67
5	1	−1.79	32	73	5	7	−1.30	37	80	0-6	1	< −1.74	< 33	< 74	5	1	−2.04	30	69
6	2	−1.6	34	76	6	10	−1.14	39	83	0-6	1	< −1.74	< 33	< 74	6	1	−1.86	31	72
7	5	−1.42	36	79	7	16	−0.98	40	85	7	1	−1.74	33	74	7	2	−1.68	33	75
8	11	−1.23	38	82	8	22	−0.81	42	88	8	1	−1.55	34	77	8	5	−1.51	35	77
9	16	−1.05	40	84	9	29	−0.65	43	90	9	4	−1.37	36	79	9	7	−1.33	37	80
10	20	−0.86	41	87	10	35	−0.49	45	93	10	9	−1.18	38	82	10	9	−1.15	38	83
11	24	−0.68	43	90	11	40	−0.33	47	95	11	17	−1.00	40	85	11	13	−0.97	40	85
12	30	−0.49	45	93	12	44	−0.16	48	98	12	23	−0.81	42	88	12	19	−0.8	42	88
13	38	−0.31	47	95	13	51	0.00	50	100	13	29	−0.63	44	91	13	25	−0.62	44	91
14	48	−0.12	49	98	14	61	0.16	52	102	14	37	−0.44	46	93	14	33	−0.44	46	93
15	58	0.06	51	101	15	67	0.33	53	105	15	46	−0.26	47	96	15	42	−0.26	47	96
16	66	0.25	52	104	16	72	0.49	55	107	16	54	−0.07	49	99	16	50	−0.09	49	99
17	73	0.43	54	106	17	79	0.65	57	110	17	62	0.11	51	102	17	56	0.09	51	101
18	79	0.62	56	109	18	83	0.81	58	112	18	68	0.30	53	104	18	64	0.27	53	104
19	83	0.8	58	112	19	87	0.98	60	115	19	72	0.48	55	107	19	72	0.45	54	107
20	84	0.99	60	115	20	89	1.14	61	117	20	76	0.67	57	110	20	78	0.62	56	109
21	87	1.17	62	118	21	90	1.30	63	120	21	80	0.85	59	113	21	84	0.80	58	112
22	89	1.35	64	120	22	92	1.46	65	122	22	84	1.04	60	116	22	88	0.98	60	115
23	92	1.54	65	123	23	94	1.63	66	124	23	87	1.22	62	118	23	90	1.16	62	117
24	95	1.72	67	126	24	95	1.79	68	127	24	89	1.41	64	121	24	93	1.33	63	120
25	97	1.91	69	129	25	97	1.95	70	129	25	92	1.59	66	124	25	95	1.51	65	123
26	98	2.09	71	131	26	97	2.11	71	132	26	95	1.78	68	127	26	96	1.69	67	125
27	98	2.28	73	134	27	97	2.28	73	134	27	96	1.96	70	129	27	96	1.87	69	128
28	98	2.46	75	137	28	98	2.44	74	137	28	97	2.15	71	132	28	97	2.04	70	131
29	98	2.65	76	140	29	98	2.60	76	139	29	98	2.33	73	135	29	98	2.22	72	133
30	98	2.83	78	143	30	99	2.76	78	141	30	98	2.52	75	138	30	99	2.40	74	136
31	98	3.02	80	145	31	99	2.93	79	144	31	98	2.71	77	141	31	99	2.58	76	139
32	98	3.20	82	148	32	99	3.09	81	146	32	99	2.89	79	143	32	99	2.75	78	141
33	98	3.39	84	151	33	99	3.25	83	149	33	99	3.08	81	146	33	99	2.93	79	144
34	99	3.57	86	154	34	99	3.42	84	151	34	99	3.26	83	149	34	99	3.11	81	147
> 34	99	> 3.57	> 86	> 154	35	99	3.58	86	154	> 34	99	> 3.26	> 83	> 149	35	99	3.29	83	149
> 34	99	> 3.57	> 86	> 154	36	99	3.74	87	156	> 34	99	> 3.26	> 83	> 149	36	99	3.46	85	152
> 34	99	> 3.57	> 86	> 154	37	99	3.90	89	159	> 34	99	> 3.26	> 83	> 149	37	99	3.64	86	155
> 34	99	> 3.57	> 86	> 154	38	99	4.07	91	161	> 34	99	> 3.26	> 83	> 149	38	99	3.82	88	157
> 34	99	> 3.57	> 86	> 154	> 38	99	> 4.07	> 91	> 161	> 34	99	> 3.26	> 83	> 149	> 38	99	> 3.82	> 88	> 157
N	64				N	109				N	204				N	136			
Mean	14.67				Mean	13.00				Mean	16.39				Mean	16.49			
SD	5.410				SD	6.149				SD	5.401				SD	5.633			
Median	14				Median	13				Median	15				Median	16			
Skewness	0.828				Skewness	0.921				Skewness	0.785				Skewness	1.371			
Kurtosis	1.488				Kurtosis	1.849				Kurtosis	0.374				Kurtosis	6.377			
Range	5-34				Range	2-38				Range	7-34				Range	4-48			

**Table 7:** Transformation of VLT-D subtest scores

**< Secondary school**										**≥ Secondary school**									
**18-39**					**40-55**					**18-39**					**40-55**				
**PD**	**P**	**z**	**T**	**IQ**	**PD**	**P**	**z**	**T**	**IQ**	**PD**	**P**	**z**	**T**	**IQ**	**PD**	**P**	**z**	**T**	**IQ**
0	2	−2.42	26	64	0	4	−1.94	31	71	0	1	−2.66	23	60	0	4	−1.94	31	71
1	4	−1.97	30	70	1	9	−1.50	35	77	1	1	−2.21	28	67	1	9	−1.5	35	77
2	7	−1.52	35	77	2	15	−1.07	39	84	2	4	−1.76	32	74	2	15	−1.07	39	84
3	14	−1.08	39	84	3	24	−0.63	44	91	3	10	−1.32	37	80	3	24	−0.63	44	91
4	24	−0.63	44	91	4	40	−0.20	48	97	4	20	−0.87	41	87	4	40	−0.2	48	97
5	41	−0.18	48	97	5	61	0.24	52	104	5	35	−0.42	46	94	5	61	0.24	52	104
6	62	0.26	53	104	6	76	0.68	57	110	6	50	0.02	50	100	6	76	0.68	57	110
7	77	0.71	57	111	7	85	1.11	61	117	7	66	0.47	55	107	7	85	1.11	61	117
8	88	1.16	62	117	8	93	1.55	65	123	8	80	0.92	59	114	8	93	1.55	65	123
9	94	1.60	66	124	9	97	1.98	70	130	9	90	1.36	64	120	9	97	1.98	70	130
10	98	2.05	71	131	10	99	2.42	74	136	10	97	1.81	68	127	10	99	2.42	74	136
N	64				N	109				N	204				N	136			
Mean	5.41				Mean	4.45				Mean	5.95				Mean	5.34			
SD	2.238				SD	2.295				SD	2.238				SD	2.401			
Median	5				Median	4				Median	6				Median	6			
Skewness	−0.153				Skewness	−0.025				Skewness	−0.156				Skewness	−0.410			
Kurtosis	0.129				Kurtosis	−0.232				Kurtosis	−0.473				Kurtosis	−0.095			
Range	0-10				Range	0-10				Range	0-10				Range	0-10			

**Table 8:** Transformation of PST subtest scores

**< Secondary school**										**≥ Secondary school**									
**18-39**					**40-55**					**18-39**					**40-55**				
**PD**	**P**	**z**	**T**	**IQ**	**PD**	**P**	**z**	**T**	**IQ**	**PD**	**P**	**z**	**T**	**IQ**	**PD**	**P**	**z**	**T**	**IQ**
0-1	1	< −2.47	< 25	< 63	0	1	−2.16	28	68	0-2	1	< −2.55	< 25	<62	0-2	1	< −1.81	< 32	< 73
0-1	1	< −2.47	< 25	< 63	1	1	−1.86	31	72	0-2	1	< −2.55	< 25	<62	0-2	1	< −1.81	< 32	< 73
2	1	−2.47	25	63	2	3	−1.57	34	76	0-2	1	< −2.55	< 25	<62	0-2	1	< −1.81	< 32	< 73
3	2	−2.12	29	68	3	10	−1.27	37	81	3	1	−2.55	25	62	3	1	−1.81	32	73
4	3	−1.77	32	73	4	20	−0.98	40	85	4	2	−2.20	28	67	4	2	−1.56	34	77
5	9	−1.42	36	79	5	29	−0.69	43	90	5	4	−1.86	31	72	5	6	−1.3	37	80
6	16	−1.07	39	84	6	38	−0.39	46	94	6	8	−1.52	35	77	6	13	−1.05	40	84
7	25	−0.72	43	89	7	49	−0.10	49	99	7	14	−1.18	38	82	7	19	−0.79	42	88
8	38	−0.38	46	94	8	58	0.20	52	103	8	20	−0.84	42	87	8	29	−0.53	45	92
9	48	−0.03	50	100	9	67	0.49	55	107	9	27	−0.50	45	92	9	40	−0.28	47	96
10	59	0.32	53	105	10	78	0.79	58	112	10	41	−0.16	48	98	10	54	−0.02	50	100
11	71	0.67	57	110	11	84	1.08	61	116	11	56	0.18	52	103	11	65	0.24	52	104
12	84	1.02	60	115	12	89	1.37	64	121	12	70	0.52	55	108	12	75	0.49	55	107
13	93	1.37	64	120	13	95	1.67	67	125	13	81	0.86	59	113	13	84	0.75	57	111
14	95	1.71	67	126	14	98	1.96	70	129	14	88	1.20	62	118	14	89	1.00	60	115
15	98	2.06	71	131	15	98	2.26	73	134	15	94	1.54	65	123	15	93	1.26	63	119
16-30	> 98	> 2.06	> 71	> 131	16	99	2.55	76	138	16	97	1.88	69	128	16	95	1.52	65	123
16-30	> 98	> 2.06	> 71	> 131	17-30	99	> 2.55	> 76	> 136	17	99	2.22	72	133	17	96	1.77	68	127
16-30	> 98	> 2.06	> 71	> 131	17-30	99	> 2.55	> 76	> 136	18-30	99	> 2.22	> 72	> 133	18	97	2.03	70	130
16-30	> 98	> 2.06	> 71	> 131	17-30	99	> 2.55	> 76	> 136	18-30	99	> 2.22	> 72	> 133	19	97	2.29	73	134
16-30	> 98	> 2.06	> 71	> 131	17-30	99	> 2.55	> 76	> 136	18-30	99	> 2.22	> 72	> 133	20	98	2.54	75	138
16-30	> 98	> 2.06	> 71	> 131	17-30	99	> 2.55	> 76	> 136	18-30	99	> 2.22	> 72	> 133	21	99	2.80	78	142
16-30	> 98	> 2.06	> 71	> 131	17-30	99	> 2.55	> 76	> 136	18-30	99	> 2.22	> 72	> 133	22	99	3.05	81	146
16-30	> 98	> 2.06	> 71	> 131	17-30	99	> 2.55	> 76	> 136	18-30	99	> 2.22	> 72	> 133	23	99	3.31	83	150
16-30	> 98	> 2.06	> 71	> 131	17-30	99	> 2.55	> 76	> 136	18-30	99	> 2.22	> 72	> 133	24	99	3.57	86	154
16-30	> 98	> 2.06	> 71	> 131	17-30	99	> 2.55	> 76	> 136	18-30	99	> 2.22	> 72	> 133	25	99	3.82	88	157
16-30	> 98	> 2.06	> 71	> 131	17-30	99	> 2.55	> 76	> 136	18-30	99	> 2.22	> 72	> 133	26	99	4.08	91	161
16-30	> 98	> 2.06	> 71	> 131	17-30	99	> 2.55	> 76	> 136	18-30	99	> 2.22	> 72	> 133	27-30	99	> 4.08	> 91	> 161
N	64				N	109				N	202				N	136			
Mean	9.08				Mean	7.33				Mean	10.48				Mean	10.08			
SD	2.869				SD	3.399				SD	2.939				SD	3.920			
Median	9				Median	7				Median	11				Median	10			
Skewness	−0.093				Skewness	0.275				Skewness	−0.194				Skewness	1.694			
Kurtosis	−0.458				Kurtosis	−0.522				Kurtosis	−0.107				Kurtosis	6.269			
Range	2-15				Range	0-16				Range	3-17				Range	3-30			

**Table 9:** Transformation of total scale scores

**< Secondary school**										**≥ Secondary school**									
**18-39**					**40-55**					**18-39**					**40-55**				
**PD**	**P**	**z**	**T**	**IQ**	**PD**	**P**	**z**	**T**	**IQ**	**PD**	**P**	**z**	**T**	**IQ**	**PD**	**P**	**z**	**T**	**IQ**
0-35	1	< −2.18	< 28	< 67	0-20	1	< −2.26	< 27	< 66	0-20	1	< −3.47	< 15	< 48	0-35	1	< −2.28	< 27	< 66
0-35	1	< −2.18	< 28	< 67	21-25	1	−2.26	27	66	21-25	1	< −3.47	< 15	< 48	0-35	1	< −2.28	< 27	< 66
0-35	1	< −2.18	< 28	< 67	26-30	2	−1.93	31	71	26-30	1	−3.47	15	48	0-35	1	< −2.28	< 27	< 66
0-35	1	< −2.18	< 28	< 67	31-35	6	−1.60	34	76	31-35	1	−3.072	19	54	0-35	1	< −2.28	< 27	< 66
36-40	1	−2.18	28	67	36-40	10	−1.27	37	81	36-40	1	−2.673	23	60	36-40	1	−2.28	27	66
41-45	2	−1.77	32	73	41-45	17	−0.93	41	86	41-45	1	−2.274	27	66	41-45	3	−1.91	31	71
46-50	7	−1.36	36	80	46-50	26	−0.60	44	91	46-50	2	−1.876	31	72	46-50	6	−1.54	35	77
51-55	19	−0.95	40	86	51-55	39	−0.27	47	96	51-55	6	−1.477	35	78	51-55	12	−1.17	38	82
56-60	33	−0.54	45	92	56-60	54	0.06	51	101	56-60	14	−1.079	39	84	56-60	21	−0.80	42	88
61-65	45	−0.13	49	98	61-65	64	0.39	54	106	61-65	25	−0.68	43	90	61-65	32	−0.43	46	94
66-70	56	0.28	53	104	66-70	76	0.72	57	111	66-70	38	−0.28	47	96	66-70	44	−0.06	49	99
71-75	67	0.69	57	110	71-75	85	1.05	61	116	71-75	53	0.12	51	102	71-75	60	0.31	53	105
76-80	82	1.09	61	116	76-80	91	1.38	64	121	76-80	66	0.52	55	108	76-80	75	0.68	57	110
81-85	93	1.50	65	123	81-85	95	1.71	67	126	81-85	79	0.91	59	114	81-85	84	1.05	60	116
86-90	99	1.91	69	129	86-90	96	2.04	70	131	86-90	89	1.31	63	120	86-90	91	1.42	64	121
> 90	99	> 1.91	> 69	> 129	91-95	98	2.37	74	136	91-95	95	1.71	67	126	91-95	95	1.79	68	127
> 90	99	> 1.91	> 69	> 129	> 96	> 98	> 2.37	> 74	> 136	96-100	99	2.11	71	132	96-100	98	2.16	72	132
> 90	99	> 1.91	> 69	> 129	> 96	> 96	> 96	> 96	> 96	> 101	99	> 2.11	> 71	> 132	101-105	99	2.53	75	138
> 90	99	> 1.91	> 69	> 129	> 96	> 96	> 96	> 96	> 96	> 101	99	> 2.11	> 71	> 132	> 105	99	> 2.53	> 75	> 138
N	64				N	109				N	202				N	134			
Mean	64.63				Mean	57.12				Mean	71.53				Mean	68.84			
SD	12.213				SD	15.109				SD	12.544				SD	13.507			
Median	64				Median	56				Median	71				Median	70			
Skewness	−0.007				Skewness	0.156				Skewness	−0.123				Skewness	−0.034			
Kurtosis	−0.890				Kurtosis	−0.124				Kurtosis	−0.218				Kurtosis	−0.244			
Range	37-88				Range	21-94				Range	30-98				Range	38-105			

After administering the instrument, for norming the cognitive performance of a patient with schizophrenia or bipolar I disorder against the reference or comparator group, the examiner has only to locate the corresponding transformed score on the table by the patient’s age and educational level.

## Discussion

The clinical value of a screening tool is directly related to either considering cognitive impairment a key aspect of schizophrenic psychopathology and, according to the proposed DSM-V revisions, recommending it as one key dimension to be measured in all patients with a psychotic disorder, or including cognitive deficit as one of the diagnostic criteria for psychoses as suggested by some authors [46,47]. The practical utility of the administered tests should not be forgotten when conducting a neuropsychological assessment, and since there is a large number of psychiatric patients (accounting for around 2% of the general population) who require diagnosis, there is a growing need for cost-effective and highly efficient diagnostic tools. In this regard, the creation of the SCIP-S precisely had these two objectives. Previous studies [33,34] have shown that the SCIP-S takes approximately 15 minutes to administer, compared to a mean of around 75 minutes for the administration of a full neuropsychological battery or between 60–90 minutes for the MCCB, and it has good validity and reliability. Furthermore, Rojo et al. [35] reported the good sensitivity and specificity of the test and its high diagnostic value for appropriately distinguishing cognitively preserved from cognitively impaired individuals.

This study goes a step farther and presents the diagnostic-specific norms and performance for the SCIP-S according to the age and educational level of subjects in a large sample of patients with schizophrenia and bipolar disorder. Some studies report differences in neuropsychological performance between subjects with different educational levels [48,49], and such differences were also found in this study, which is why in the SCIP-S standardization data the sample has been divided based on educational level. It should be pointed out that, although there is not always a direct correspondence between educational level and years of education, we may consider that in the vast majority of cases a less than secondary education implies fewer than 12 years of education, while a secondary or higher educational level implies at least 12 years of education.

Another aspect known to influence cognitive performance is age, as over the years a series of cortical changes occurs resulting in a loss of brain volume [50,51] associated with a decrease in cognitive performance in the general population [52,53]. Our norms take this aspect into account by dividing the sample according to age and limiting patient age to 55 years in order not to introduce bias due to patients whose performance could be affected by early onset of a picture of dementia.

One item that bears emphasizing refers to that fact that a certain pattern was observed to repeat in the various subtests and total SCIP-S score. Specifically, the median score in the two age groups is similar (a maximum difference of 1 point) when the patients have a high educational level, while differences of up to 8 points are found in the groups with a primary or lower educational level. This may be explained by an interaction between age and educational level and the possibility that age at onset of illness plays an important role, since some studies show that both verbal intelligence and impairment of verbal memory and executive functioning could be affected in patients before they experience their first psychotic episode [54-56], such that the earlier the onset of illness, the greater the potential for limiting the patient’s ability to normally pursue an education. Therefore, the effects of age and educational level and their interaction were explored by adding age at onset of illness as a covariate. Such interaction was not statistically significant in any SCIP-S score (all *p* > .01).

One of the aspects highlighted by this study is that of all the tests mentioned above that have been developed for the purposes of cognitively assessing psychiatric patients, we find diagnosis-specific standardization data for patients with schizophrenia only for the RBANS [39,40]. And as stated by Iverson et al. [40], being able to describe a patient’s cognitive performance in terms of expectation for their peer group is more useful to multidisciplinary treatment teams than just comparing them to a healthy population. So the present study provides the tools necessary to interpret the score obtained by a patient with functional psychosis relative to other patients after administration of the SCIP-S scale. As an example, let us apply the SCIP-S to an imaginary 38 year old college graduate diagnosed with schizophrenia who obtains a direct score of 13 on the WMT subtest. After determining their performance relative to healthy controls (healthy control norms are under elaboration), the clinician interest could move to answer the question: Where do we situate that individual with respect to other patients? Looking at Table 4, we see that, based on his age and educational level, this patient is in the 9th percentile, has a z-score of −1.45, a T-score of 36, and an IQ of 78. This tells us that only 9% of his comparison group has obtained a score below his and that his working memory score of 13 is situated approximately 1.5 standard deviations below the other patients.

## Conclusions

The SCIP and the SCIP-S provides a quick and convenient cognitive diagnosis and, in that regard, its usefulness extends to areas such as detection, cognitive assessment of large samples, epidemiological and screening diagnostic studies more than to specific cognitive domains or type of impairment in patients with functional psychosis. In that way, it is a complementary test that is not intended to replace complete neuropsychological batteries. Future studies should explore how performance on the SCIP relates to other cognitive domains that it does not measure directly (e.g., problem solving, social cognition, etc.).

A study that could continue this one should be perform standardization data for patients over 55 years of age, since, at a cognitive level, that is a critical age where the SCIP-S could help us reach a differential diagnosis between onset of dementia versus cognitive dysfunction associated with functional psychosis. Future research with this scale should also incorporate the development of guidelines for interpreting the scoring according to results of treatment of patients. Additionally, last evidences in schizophrenic and bipolar patients from recent studies have suggested that the history of psychosis explain part of the neurocognitive performance [57], thus in future studies with cognitive screening tools would be interesting to take this variable into account.

In short, this study presents the first jointly developed diagnostic-specific norms for the SCIP for functional psychosis (schizophrenia and bipolar disorder), providing increased information about their cognitive abilities.

## Competing interests

Javier Rejas and Francisco Mesa are employees of Pfizer Spain, the body funding the original study sourcing data for this manuscript. All other authors declare that they have no conflicts of interests and none received payments or honoraria as a consequence of authorship for this manuscript.

## Authors’ contributions

This was a collaborative work, and the authors worked closely each other. All authors participated in the design of the original study or in the interpretation and analysis of data and all of them drafting and have approved the final version of manuscript. All authors were responsible for literature review and extraction of references, and also for taking the decision to submit the paper for publication.

## Pre-publication history

The pre-publication history for this paper can be accessed here:

http://www.biomedcentral.com/1471-244X/13/127/prepub
